# Sign language instrument for assessing the knowledge of deaf people about Cardiopulmonary Resuscitation*

**DOI:** 10.1590/1518-8345.3535.3283

**Published:** 2020-06-08

**Authors:** Nelson Miguel Galindo-Neto, Magno Batista Lima, Lívia Moreira Barros, Silvana Cavalcanti dos Santos, Joselany Áfio Caetano

**Affiliations:** 1 Instituto Federal de Educação, Ciência e Tecnologia de Pernambuco (IFPE), Campus Pesqueira, Pesqueira, PE, Brazil.; 2 Universidade Federal do Piauí, Colégio Técnico de Bom Jesus, Bom Jesus, PI, Brazil.; 3 Universidade Estadual Vale do Acaraú, Departamento de Enfermagem, Sobral, CE, Brazil.; 4 Universidade Federal do Ceará, Departamento de Enfermagem, Fortaleza, CE, Brazil.

**Keywords:** Persons with Hearing Impairments, Sign Language, Cardiopulmonary Resuscitation, Knowledge, Validation Studies, Health Education, Pessoas com Deficiência Auditiva, Linguagem de Sinais, Reanimação Cardiopulmonar, Conhecimento, Estudos de Validação, Educação em Saúde, Personas com Deficiencia Auditiva, Lengua de Signos, Reanimación Cardiopulmonar, Conocimiento, Estudios de Validación, Educación em Salud

## Abstract

**Objective:**

to build and validate the content on Cardiopulmonary Resuscitation (CPR) of a sign language instrument for assessing the knowledge of the deaf.

**Method:**

methodological study in which the content validity process was used by 22 specialists in cardiac arrest and 16 deaf people. In the validation of internal consistency, 113 deaf people participated. For the assessment of the deaf, the Assistive Technology Assessment Questionnaire was used and, in the content validity, an instrument with a Likert scale was used, which included the content, clarity, objectivity, organization and language. Items with a minimum agreement of 80% were considered valid, according to the Content Validity Index (CVI) and binomial test. The internal consistency was verified by Cronbach’s alpha.

**Results:**

The instrument contains 11 questions about the identification of cardiorespiratory arrest, activation by aid and high quality chest compression. It had a minimum content validity of 81% by the specialists, 90% by the deaf participants and internal consistency by the Cronbach alpha of 0.86, being considered high.

**Conclusion:**

the instrument can be used in research to survey the previous knowledge of deaf people about CPR, as well as in pre and/or post-testing studies that test educational interventions with this public.

## Abstract (pt)

**Objetivo:**

construir e validar o conteúdo sobre Ressuscitação Cardiopulmonar (RCP) de instrumento em língua de sinais para a avaliação do conhecimento de surdos.

**Método:**

estudo metodológico no qual o processo de validação de conteúdo foi utilizado por 22 especialistas em parada cardiorrespiratória e por 16 surdos. Na validação da consistência interna, participaram 113 surdos. Para a avaliação dos surdos, foi utilizado o Questionário de Avaliação de Tecnologia Assistiva e, na validação de conteúdo, foi utilizado instrumento com escala tipo Likert, que contemplou o conteúdo, a clareza, a objetividade, a organização e a linguagem. Foram considerados válidos os itens com concordância mínima de 80%, conforme o Índice de Validação de Conteúdo e teste binomial. A consistência interna foi verificada pelo alpha de Cronbach.

**Resultados:**

o instrumento contém 11 questões sobre a identificação da parada cardiorrespiratória, o acionamento por ajuda e as compressões torácicas de alta qualidade. Apresentou validade de conteúdo mínima de 81% pelos especialistas, de 90% pelos participantes surdos e consistência interna pelo alpha de Cronbach de 0,86, sendo considerada alta.

**Conclusão:**

o instrumento pode ser utilizado em pesquisas para levantamento do conhecimento prévio de surdos acerca da RCP, bem como em pré e/ou pós-teste de estudos que testem intervenções educativas com esse público.

## Abstract (es)

**Objetivo:**

construir y validar el contenido sobre Reanimación Cardiopulmonar (RCP) de instrumento en lenguaje de señales para evaluación del conocimiento de sordos.

**Método:**

estudio metodológico, en el cual el proceso de validación de contenido tuvo como participantes por 22 especialistas en parada cardiorrespiratoria y por 16 sordos. En la validación de la consistencia interna participaron 113 sordos. Para la evaluación de los sordos fue utilizado el Cuestionario de Evaluación de Tecnología Asistencial y en la validación de contenido fue utilizado un instrumento con escala tipo Likert, que contempló contenido, clareza, objetividad, organización y lenguaje. Fueron considerados válidos los ítems con concordancia mínima de 80%, conforme el Índice de Validación de Contenido y test binomial. La consistencia interna fue verificada por el alpha de Cronbach.

**Resultados:**

el instrumento contiene 11 preguntas sobre: la identificación de la parada cardiorrespiratoria, el accionamiento por ayuda y las compresiones torácicas de alta calidad. Este presentó validez de contenido mínima de 81% por los especialistas, de 90% por los participantes sordos y consistencia interna por el alpha de Cronbach de 0,86, la que fue considerada alta.

**Conclusión:**

el instrumento puede ser utilizado en investigaciones para levantamiento del conocimiento previo de sordos acerca de la RCP, así como en pretest y/o postest de estudios que comprueban intervenciones educativas con ese público.

## Introduction

The deaf constitute a category of the world’s population of some 466 million people, estimated by the World Health Organization to reach 900 million by 2050^(^ 1 ^)^. Communication of the deaf, when hearing impairment is greater than 40 dB, occurs by means of a gesture stimulus^(^ 2 ^)^, Thus, this public has difficulty in accessing information on various health-related topics^(^ 3 ^)^.

The communication barrier makes it impossible to empower deaf people about life-threatening health situations for which they could know how to act and contribute to reducing mortality. An addiction incompatible with life and in which the intervention of people who are not health professionals is associated with greater survival is out-of-hospital cardiac arrest (CRA)^(^ 4 ^)^. It affects almost 300,000 people a year in the United States^(^ 5 ^)^ and 100,000 in Brazil^(^ 6 ^)^.

To assist victims of CRA, all individuals with cognitive conditions to perform their identification and motor skills to perform chest compressions should be taught on this subject^(^ 4 ^)^. One of these audiences is composed of the deaf, who may be present in various social environments and witness a CRA outside the hospital, so that the need for investment in research that takes into account the specificities of teaching cardiopulmonary resuscitation (CPR) to this part of the population is highlighted.

In order to make scientific research on the teaching of CPR for the deaf possible, it is pertinent to evaluate the knowledge before and after educational interventions. In order to deal with internal validity, such studies should carry out measurements of the knowledge of the deaf about CPR from instruments built and validated.

Nursing is the professional category that has health education present in its professional exercise^(^ 7 ^)^. Therefore, as it has expertise in emergency related issues, it constitutes a strategic professional category for health education for deaf people on CPR. Thus, it is important to note that research that includes tools to assess knowledge of deaf people about CPR is important for such a category, which may use such tools in studies that test various interventions and teaching strategies in teaching CPR to deaf people. In addition, this tool could be used in institutions teaching deaf people even in the absence of the professional nurse.

Thus, the study aimed to build and validate the content on Cardiopulmonary Resuscitation of a sign language instrument for the assessment of the knowledge of deaf people.

## Method

Methodological research, operated according to psychometry, from the theoretical, empirical and analytical poles^(^ 8 ^-^ 9 ^)^. The theoretical pole corresponded to the first stage: the construction of the instrument. The empirical pole consisted of the second stage, of content validity by 22 professionals, the third stage, of validation by 16 deaf people, and the fourth stage, of application with 113 other deaf people to verify internal consistency. In the analytical stage, content validity and reliability were calculated/verified.

The study was carried out in three educational institutions located in Fortaleza - CE. The first two were state public schools: one dedicated exclusively to the education of the deaf and the second dedicated to the teaching of the deaf and hearing. The third institution was philanthropic, religious and aimed at teaching and social inclusion of the deaf.

In the first stage, construction of the instrument, questions were elaborated based on the recommendations for lay people from the Brazilian Society of Cardiology, American Health Association, Resuscitation Councils of Asia and Europe regarding the way to identify the CRA, the need to call for help and the correct chest compression^(^ 10 ^-^ 13 ^)^. The distribution of the themes for each question is summarized in Figure 1.

**Figure 1:** Themes of the instrument questions to assess the knowledge of the deaf about Cardiopulmonary Resuscitation. Fortaleza, CE, Brazil, 2018

Block of questions	Theme of each question
Identify	1 - Correctly identify cardiorespiratory arrest with verification of the victim’s responsiveness and breathing.
	2 - Emergence and severity of cardiorespiratory arrest.
Call for help	3 - Calling for help before starting chest compressions.
	4 - Telephone contact number with the Mobile Emergency Care Service (MECS).
Chest compression	5 - Correct moment when chest compression should be performed.
	6 - Position the victim correctly in a rigid and level place.
	7 - Place hands in the center of the victim’s chest for compression.
	8 - Position shoulders at 90º with the victim’s chest.
	9 - Perform chest compression with as much force as possible.
	10 - Relay the rescuer who compresses every two minutes.
	11 - Do not stop compressions until the victim wakes up or until health professionals arrive.

To expand the possibility of understanding the instrument, the questions were built by a team composed of a nurse with experience in urgency and emergency and in the teaching of CRA, by a Libras interpreter and by a teacher from a deaf education institution fluent in Libras. Both, in a face-to-face meeting, formulated the instrument’s 11 questions, as well as the five alternatives for each one.

Each question was composed of five answer options (multiple choice), with only one correct alternative, a clear and concise statement, which requested the correct alternative related to the competence/information evaluated. The alternatives that were not correct were built from possible situations so that the correctness would not occur by the logical selection of the alternatives, except the last alternative for each question (the letter “e”), which was standardized with the option “I don’t know”, which could be marked by the deaf.

In two questions (referring to the location of the rescuer’s position and hands for chest compression), the alternatives had images to facilitate their understanding. In the alternatives of all questions, the letters a, b, c, d and e were accompanied by images of hands with that letter of the alphabet in Libras. It should be noted that all images of the instrument were built by a professional designer, hired exclusively for this purpose, using the Corel Draw program.

In the second stage, content validation, the instrument was submitted to content validation by experts in CRA. The definition of the sample for this step occurred from the formula for the finite population $n={\mathrm{Za}}^{2}.P\left(1-P\right)/e^{2}$ ^(^ 14 ^)^. The confidence level (Za) used was 95%; the proportion of expert agreement (P) used was 85% and the acceptable difference (e) used was 15%. Using these parameters, we obtained the sample quantity of 22.

Professionals with expertise in CRA were recruited from the websites of the public institutions of higher education in Fortaleza - CE where the collection of e-mail addresses of teachers in the emergency and intensive care areas of the undergraduate courses in Nursing took place. Through e-mail contact with such professionals, there was a request for nominations of other professionals (snowball strategy) so that indications of names of professionals from the states of Rio de Janeiro, São Paulo, Minas Gerais, Paraíba and Pernambuco were obtained.

The criteria for the selection of specialists dealt with academic, scientific and professional aspects^(^ 15 ^)^: were used as inclusion criteria to be a bachelor in Nursing and to have teaching, assistance or CRA research experience. The exclusion criterion was the non-fulfillment of the data collection instrument. Thus, 54 professionals were contacted by e-mail to whom the Term of Free and Informed Consent (FICT), the instrument in the validation process and the instrument for data collection were sent. Thus, the 11 questions of the instrument in process of validation were analyzed by the professionals, who filled in the data collection instrument regarding its evaluation/validation, composed of 14 questions for the characterization of the specialists and eight questions, on a Likert type scale, for the professional to record his level of agreement about the scope, relevance and clarity of the content, objectivity, organization and language.

For the registration of the experts’ agreement, the instrument had the options 1 = I totally disagree, 2 = I neither agree nor disagree, 3 = I partially agree and 4 = I totally agree. In addition, for each question evaluated, space was made available for the registration of opinions/suggestions.

The 22 professionals who first sent the response were included in the sample of this phase of the survey. After analyzing the suggestions obtained, the recommendation of reformulating three sentences was followed to make the alternatives more understandable.

As it was an instrument aimed at the deaf, the questions were recorded in studio in the Brazilian Sign Language (Libras) by a duly certified professional (holder of a diploma of technical course in interpreting Libras), who worked as an interpreter in one of the institutions for teaching deaf people. Thus, the evaluation instrument was composed of two parts: the first in written/printed Portuguese and the second in Libras, in video format. Both to be used concomitantly in the evaluation of deaf people’s knowledge about CPR: the video for the questions and alternatives in Libras to be watched and the printed part for recording the answers.

In the third stage, validation by the deaf, both parts of the instrument (printed and video) were evaluated by 16 participants in a professional course in a philanthropic institution. This institution, with about 150 deaf students enrolled, had a teaching offer from the first to the ninth year, in addition to offering a professional course in administrative assistant. The sample quantity of this stage was defined for convenience: through prior contact with the coordination of the institution, it recommended the evaluation of the instruments by the students of the vocational course in view of the availability of such class.

The inclusion criterion for the participants of this stage was to have a link as a student of the institution and the exclusion criterion was to have cognitive impairment that made it impossible to judge the material. This commitment was verified by consulting the medical reports, existing in the school office, which were a requirement for the registration of students. These reports included medical records regarding deafness, presence of comorbidities and cognitive impairment.

The communication process with deaf students was carried out by the presence of a Libras interpreter, from the said educational institution, who asked the participants to sign the FICT. The participants were accommodated in the classroom and seated in school desks in a semicircle. For each one, the printed version of the instrument was delivered and the video was projected to the center of the semicircle in a date show. Each question was projected on video and, after exposing the statement and the five alternatives in the video, the students were invited by the interpreter to read the same question in the printed part of the instrument. Thus, there was video exposure and reading of the 11 questions of the instrument.

After the presentation of all questions, to record the evaluation of deaf students, the participants were given the Assistive Technology Assessment Questionnaire (ATAQ). This questionnaire, constructed and validated for people with disabilities to assess their understanding of assistive technologies, has 14 questions about the objective, clarity, relevance and interactivity for which it is possible for the participant to mark, on the response scale, the “inappropriate”, “partially appropriate” or “appropriate” options for each item assessed^(^ 16 ^)^. For the use of ATAQ, each question was read by the researcher, with concomitant translation into Libras by the interpreter. This reading/translation took place in one question at a time and after each question was read, the deaf were asked to fill out the answer.

In the fourth step, to check the internal consistency, the instrument was applied to the deaf. For this, there was the scheduling with the coordination of the three educational institutions that had deaf students in Fortaleza - CE. The classes of the three shifts were approached in their class schedule with the authorization of the teacher of the referred schedule.

The inclusion criterion was to be duly enrolled in the educational institution and the exclusion criteria were: not having attended classes at the educational institution, so that the participant was not present on the day scheduled for data collection, and being affected by cognitive impairment that made participation in the research impossible. This commitment was verified by consulting the existing medical reports in the secretariats of educational institutions.

Of the 360 deaf students enrolled in the three institutions, 247 were excluded because they were not present on the day scheduled for data collection. Thus, 113 integrated the sample of this stage. It should be noted that this quantitative is compatible with the sample to determine internal consistency, which should be from five to ten participants *per* item^(^ 17 ^)^. Thus, considering that the instrument has 11 items, the sample to estimate its internal consistency would be from 55 to 220 participants.

The application of the instrument was mediated by a Libras interpreter. The FICT was requested to be signed and, with the participants accommodated in their respective school desks, the printed instrument was delivered. The video part of the instrument was projected on the whiteboard located in front of the room. Each question was contemplated, in turn, so that the projection of the question/answer and five alternatives took place, with subsequent provision of time for the recording of the answer in the printed part of the instrument.

In the analytic pole, the data were analyzed in the R software, version 3.1.1. In the content validation with the professionals, the Content Validity Index (CVI) was used, which was calculated, for each item, from the amount of experts who agreed with the item, sum of the answers 3 = partially agree and 4 = fully agree, divided by the total amount of experts. In content validation with deaf people, since the questionnaire used, ATAQ, had the response options inadequate, partially adequate and adequate, the CVI was calculated, per item, from the quantity of deaf people who considered the adequate item divided by the total number of deaf people who validated the material.

For the analysis about the proportion of agreement having been equal or superior to 80%, value determined to consider the valid item^(^ 18 ^)^, the binomial test was used. The reliability of the instrument was verified through internal consistency from the Cronbach alpha calculation. For Cronbach’s alpha classification, which varies from 0 to 1, it was considered very low for values below 0.30; low for values between 0.3 and 0.6; moderate for values above 0.6 and below 0.75; high for values between 0.75 and 0.9 and very high for values above 0.9^(^ 17 ^)^.

The research was conducted in accordance with Resolution 466/12 and obtained approval from the Research Ethics Committee of the Federal University of Ceará according to opinion 2,108,475.

## Results

**Figure 2:**
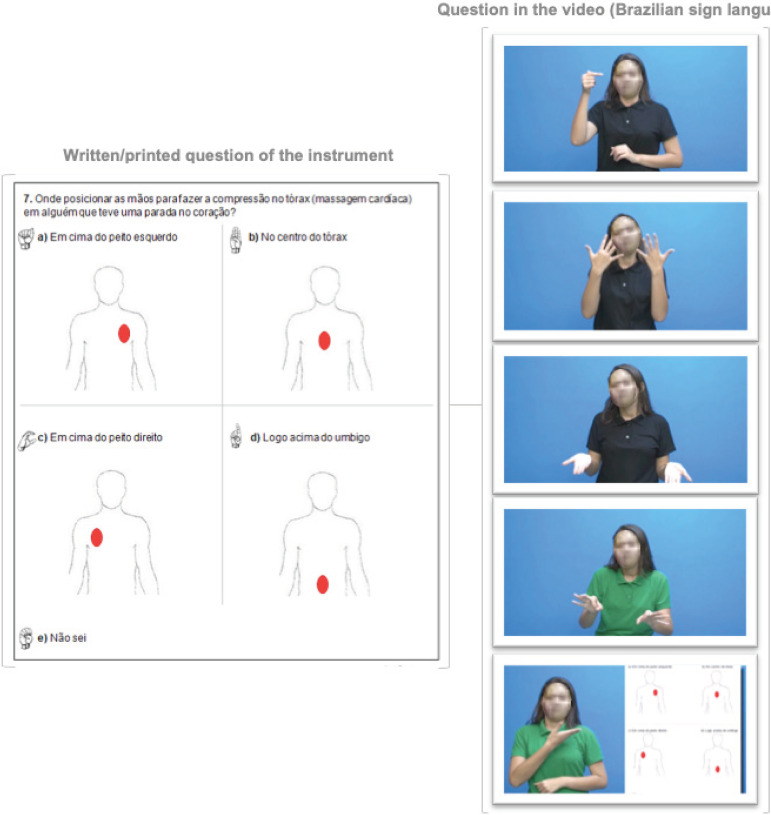
Question of the instrument to evaluate the knowledge of deaf people on Cardiopulmonary Resuscitation. Fortaleza, CE, Brazil, 2018

The instrument had 11 questions that included the identification of CRA, the triggering of aid and the performance of CPR. In addition, it was composed of two parts: the first in video, with the questions and alternatives in Libras and the second printed, with the written content for recording the answer. In the video part, in a standard way, the interpreter presented herself with a black blouse during the narration of all the questions and with a green blouse in the narratives of the answer alternatives. Figure 2 shows how the same question was addressed in the video (Libras) and printed parts.

**Figure 3:**
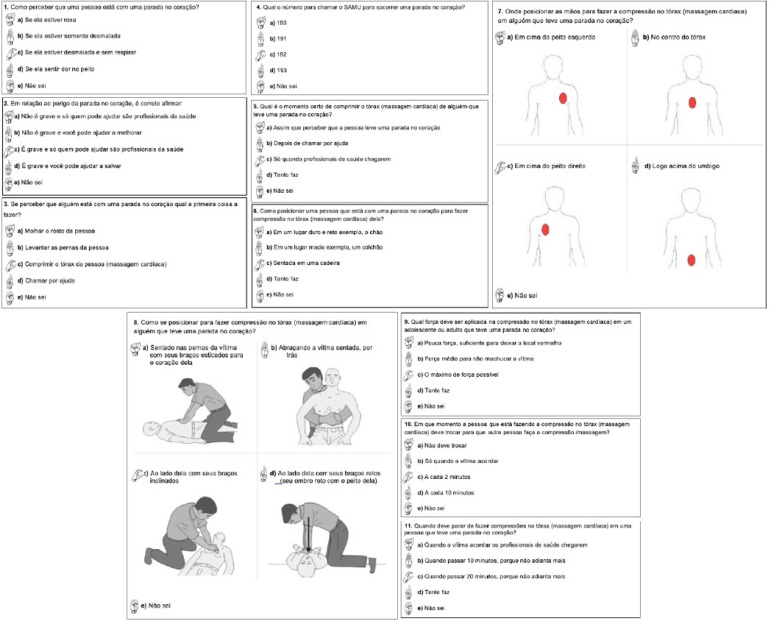
Pages of the instrument to evaluate the knowledge of the deaf on Cardiopulmonary Resuscitation. Fortaleza, CE, Brazil, 2018

The video version of the instrument was finished with a duration of ten minutes and 50 seconds, while the printed/written version was composed of five pages. The pages of the printed part of the instrument and the questions and alternatives that compose it can be seen in Figure 3.

The specialists who participated in the validity of the contents of the instrument were all nurses, being nine (40.9%) doctors, eight (36.4%) masters and five (22.7%) specialists. The 22 professionals had assistance experience in the areas of Emergency and Emergency or Intensive Care and had participated in training courses on CPR. In addition, 19 (86.3%) were teachers of higher education or specialization courses and taught curricular components related to CRA.

The content validation pointed out agreement higher than 80% in all questions and, due to the non-significance of the binomial test, all questions showed agreement statistically higher than 0.8. Thus, all questions were considered valid in terms of content by the experts (Table 1).

**Table 1:** Experts agreement about the instrument to assess the knowledge of deaf people on Cardiopulmonary Resuscitation. Fortaleza, CE, Brazil, 2018

Question	Content		Clarity		Objectivity		Organization		Language	
	CVI*	p^†^	CVI	p	CVI	p	CVI	p	CVI	p
**1**	0.81	0.424	0.81	0.42	0.95	0.972	0.86	0.661	0.90	0.863
**2**	1	1	0.86	0.66	0.95	0.972	0.86	0.661	0.95	0.972
**3**	0.95	0.972	1	1	1	1	0.90	0.863	0.95	0.972
**4**	1	1	1	1	1	1	1	1	1	1
**5**	0.95	0.972	0.86	0.66	0.95	0.972	0.86	0.661	0.95	0.972
**6**	1	1	1	1	1	1	0.95	0.972	0.95	0.972
**7**	1	1	1	1	1	1	1	1	0.95	0.972
**8**	1	1	1	1	1	1	0.95	0.972	0.90	0.863
**9**	0.86	0.661	0.81	0.42	0.86	0.661	0.86	0.661	0.95	0.972
**10**	0.95	0.972	0.95	0.97	1	1	1	1	0.90	0.863
**11**	1	1	1	1	1	1	1	1	1	1

* CVI = Content Validity Index;

† p = Binomial test

Regarding the deaf who evaluated the instrument, 13 (81.2%) were female; ten (62.5%) were married and nine (56.2%) had no children. According to the assessment of the deaf, the questions of the instrument (statements and alternatives) were considered understandable by all. There was unanimous agreement (with binomial test = 1) on questions about the suitability of using the material, ease and encouragement for use, encouragement for learning, attractiveness, clarity and encouragement for reflection. The only item in which there was no total agreement was about the material enabling interaction, which obtained 95% agreement for the deaf and whose binomial test was 0.972. It is noteworthy that the deaf did not present any suggestion of adjustment / improvement for the instrument.

The instrument’s reliability was attested by the high internal consistency observed from Cronbach’s alpha equal to 0.82.

## Discussion

The constructs addressed by the instrument questions included three stages of assistance: the identification of CRA, the correct activation by help and the performance of CPR. This finding corroborates a study carried out in Spain, which built and validated a tool for the evaluation of the quality of CPR and obtained, from specialist professionals, the recommendation on the relevance of evaluating the identification of CRA, the activation by help and the performance of CPR^(^ 19 ^)^. Such findings converge with a Brazilian study, which dealt with the construction and validation of educational technology for deaf people about CPR, in which the same steps of assistance were addressed^(^ 20 ^)^. Thus, the relevance of these three constructs to be addressed in the instrument’s questions is highlighted.

The identification of the CRA and the call for help are ratified by the fact that the assistance of health professionals depends on someone to identify the problem and inform the health team of the situation^(^ 11 ^,^ 21 ^)^. While the high quality of CPR, composed of the correct positioning of the victim and the rescuer, the position of the hands in the center of the chest, the correct speed and depth, the minimization of interruptions and the return of the chest between compressions, impacts the maintenance of perfusion blood flow and the return of spontaneous circulation, as pointed out by an American multicenter study^(^ 21 ^)^.

The process of selecting the themes of the questions corroborated the positive evaluation of the experts, as there was agreement that the content was correct and sufficient to assess the necessary dimensions of the theme. Such agreement converges with that found in a study from Venezuela, which contemplated the construction and validation of educational technology about oral health^(^ 22 ^)^. The findings mentioned above confirm the need for evaluation / validation of instruments by specialists before being used in research.

The construction of the instrument aimed at the deaf demanded attention to the communication specificities of this population. About this, results of research carried out in South Africa showed that the deaf point to the use of sign language and images as factors that facilitate their understanding^(^ 2 ^)^. This finding confirms the recommendations of professionals working in the education of the deaf about the need for communication to occur from visual stimuli (images) and sign language and, when writing is necessary, occur with simple concepts and short texts^(^ 23 ^)^. The caution with writing is due to the linguistic barrier of the deaf with writing, which causes difficulty in understanding^(^ 24 ^)^.

In this way, the instrument is supported by having the letters of the alternative answers (a, b, c, d and e) accompanied by an image of that letter in Libras, consisting of two parts (one on video/Libras, for effective communication and the other written/printed to record responses) and have your text prepared with the help of an interpreter and deaf teacher.

The fulfillment of the specifics of communication with the deaf reflected in the experts’ agreement about the clarity, objectivity, organization and language of the instrument. Such agreement is similar to that found in a study that evaluated video aimed at teaching the human genome conducted in New York^(^ 25 ^)^. In this context, it is noteworthy that the process of communication between professionals and the population, in instruments, requires caution, organization and adaptation of technical terms so that the data from their use are not biased and, therefore, do not compromise quality and feasibility of use.

The assessment of the instrument by deaf people was relevant in view of the possibility of obtaining suggestions regarding the reformulation of confusing or poorly understood text, images or text.

The difficulty in understanding the population, regarding health content, is verified in two studies: the first, carried out in the United States, pointed out that 80% of the virtual page content was not understood by patients^(^ 26 ^)^. The second, from Germany, showed that the target population (patients) did not understand the content of educational materials related to ophthalmology, which were distributed/used in 32 hospitals^(^ 27 ^)^.

The aforementioned results diverge from Brazilian research on instrument validation for assessing the knowledge of adolescents about leprosy, whose results showed that the target audience considered the instrument understandable^(^ 28 ^)^.

Given the dichotomy of the results of the studies, the relevance of, in the process of validating the content of instruments, highlighting the opinion of the target audience for whom the instrument is intended is highlighted. The importance of popular participation in the evaluation of instruments is pointed out in a study carried out in Puerto Rico, which involved members of the school community in the validation of an instrument on school violence. The results of this study showed that the participation of the target audience provided important information for researchers and empowered the community to recognize their role in solving the problem^(^ 29 ^)^.

The deaf considered the instrument to be easy to use and attractive. This finding converges with that found in a study in which technology for teaching sign language was conducted in Bogotá^(^ 30 ^)^. Thus, it points out the need for instruments aimed at the deaf to be attractive and easy to use in order to make the information collection process carried out through its use feasible.

The study’s limitation refers to the fact that it was carried out in educational institutions for the deaf so that the findings may differ from research carried out with deaf people not included in the school context.

The construction and validation of the sign language instrument for assessing the knowledge of the deaf about Cardiopulmonary Resuscitation contributes to the advancement of scientific knowledge due to the availability of an instrument whose construction was carried out in a multidisciplinary way (by a nurse, Libras interpreter and teacher of deaf), based on research steps, from which it was possible to verify its content validity, internal consistency and understanding of the target audience. Thus, the instrument can be used in teaching, research and extension about the knowledge of deaf people about CPR.

Nursing acts in health education, in the care of the deaf population, in research on the knowledge of the population and in health services in which CRA occurs. Therefore, this professional category is in a strategic position to use the instrument on the knowledge of deaf people about CPR. In addition, this study presents a method that can be replicated in nursing research that contemplates the construction and validation of instruments for assessing the knowledge of deaf people about other health topics.

## Conclusion

There was the construction of the instrument in sign language to assess the knowledge of deaf people about Cardiopulmonary Resuscitation. It was composed of two parts: the first in video/Libras to establish better communication and the second written/printed to record responses. Both parties had the same questions, which included the stages of identification of the CRA, activation by help and high-quality chest compressions.

The sign language instrument for assessing the knowledge of deaf people about Cardiopulmonary Resuscitation had content validity by specialists and deaf participants and high internal consistency by Cronbach’s alpha. This instrument can be used in research to survey the deaf’s prior knowledge about CPR, as well as in pre and / or post-test studies that test educational interventions with this audience.
